# In vitro model of predicting metastatic ability using tumor derived extracellular vesicles; beyond seed soil hypothesis

**DOI:** 10.1038/s41598-022-24443-8

**Published:** 2022-11-24

**Authors:** Kinjal Bhadresha, Vinal Upadhyay, Jpan Brahmbhatt, Muhammad Jameel Mughal, Nayan Jain, Rakesh Rawal

**Affiliations:** 1 Department of Life Sciences, School of Sciences, Gujarat University, Ahmedabad, Gujarat India; 2 Hematology/Oncology Division, School of Medicine, Indiana University, Indianapolis, IN USA; 3 Department of Biochemistry and Molecular Medicine, School of Medicine and Health Science, The George Washington University, Washington, DC USA

**Keywords:** Cancer, Biomarkers

## Abstract

Lung cancer progression is often driven by metastasis, which has resulted in a considerable increase in lung cancer-related deaths. Cell-derived extracellular vesicles (EVs), particularly exosomes, serve key roles in cellular signal transmission via microenvironment, however, their biological relevance in cancer development and metastasis still needs to be clear. Here, we demonstrate that extracellular vesicles (EVs) derived from lung cancer bone metastatic patients exhibited a great capacity to promote the progression of lung cancer cells. We carried out a comprehensive meta-analysis to identify the gene expression profile of bone metastases using publicly available microarray datasets. Furthermore, mRNA expression of six identified genes was quantified by real time PCR in lung cancer with and without bone metastasis and healthy individual derived EVs. In addition, we utilized a very novel approach by to study how lung cancer cells uptake EVs by co-culturing EVs with lung cells. We observed that EVs obtained from bone metastases patients were efficiently ingested by lung cancer cells. Morevore, integration and uptake of these EVs lead to increased lung cancer cell proliferation, migration, invasion, and sphere formation. We discovered that EV uptake increase the expression of *SPP1*, *CD44*, and *POSTN* genes in lung cancer cells. The data obtained from this study, support to the possibility that circulating EVs play a significant role in the formation of the pre-metastatic niche, eventually leading to metastasis.

## Introduction

The most common cancer in the world and the one with the highest rates of morbidity and mortality is lung cancer. Its five-year survival rate after diagnosis is only 15.9%, and it has not increased in decades^1,2^. The most frequent invasive development of lung cancer is metastasis, which is one of the main reasons for death, including metastasis to the liver, bone, and leptomeninges^3^. Bone is the most frequent site of distant metastasis in lung cancer, with an incidence ranging from 30 to 40%^4^. In addition, the median survival period of patients with bone metastasis is frequently less than 1 year from diagnosis of bone metastasis to death^5^. Though the survival rate is notably increasing after neoadjuvant and surgical treatment, patients with metastatic disease still have a poor prognosis, particularly those with bone metastasis^6,7^. Moreover, various studies indicated that bone metastasis conclusively affects overall lung cancer survival, but the risk factors related to bone metastasis in patients with lung cancer remain unclear^8^. Therefore, early diagnosis and prevention stragaties of bone metastasis are clinically important to increase the survival rate of lung cancer patients.

Exosomes, microvesicles, ectosomes, and other cell-derived vesicles are examples of extracellular vesicles (EVs) found in the microenvironment. According to MISEV2018, EVs can be classified based on their physical characteristics, such as small EVs and medium/large EVs^9^. EVs released by multivesicular bodies are typically released into the extracellular matrix by cytomembrane fusion and carry a variety of cargoes, such as proteins, lipids, and nucleic acid^10,11^. Furthermore, a recent study showed that EVs play crucial roles in cellular signaling transduction as well as intercellular communication^12^. In addition, EVs may induce alteration in the tumor microenvironment and promote cancer metastasis and progression^13^. EVs that contain nucleic acids have been investigated for their role in cellular immunomodulation, resistance to chemotherapy, and cancer progression. These include miRNA, mRNA, and DNA fragments^14^. Studies have shown that Wnt3a/β-catenin, circSATB2, HIF-1α/COX-2, KLF9, and LMO7 in tumor-derived exosomes regulate genes or signaling pathways to stimulate the proliferation and migration of lung cancer cells^15–17^. Therefore, EVs cargo characterization is at the center stage of cancer research with abundant potential for noninvasive diagnosis, prognosis, and therapeutic monitoring. However, its role in the development and progression of bone metastasis in lung cancer has been less explored.

In the present study, we explored the potential role of EVs in the development of bone metastasis in lung cancer using an In-vitro model. In order to predict bone metastesis, we first established a metastatic gene signature panel using publicly available microarray-based datasets of the major  cancer types with and without bone metastasis, and then validated the result in  patient-derived EVs. Additionally, we developed a cancer microenvironment model by co-culturing A549 lung cancer cell line with serum derived EVs from lung cancer bone metastesis patients. Finally, we looked into how these isolated EVs affected the A549 cell lines ability to migrate, invade, and alter the cell cycle, as well as engage in a number of  downstream signaling pathways. Our findings suggest that EVs derived from patient serum may offer a useful way to monitor the development and spread of lung cancer bone metastasis.

## Results

### Identification of DEGs in bone metastasis

**Figure 1:**
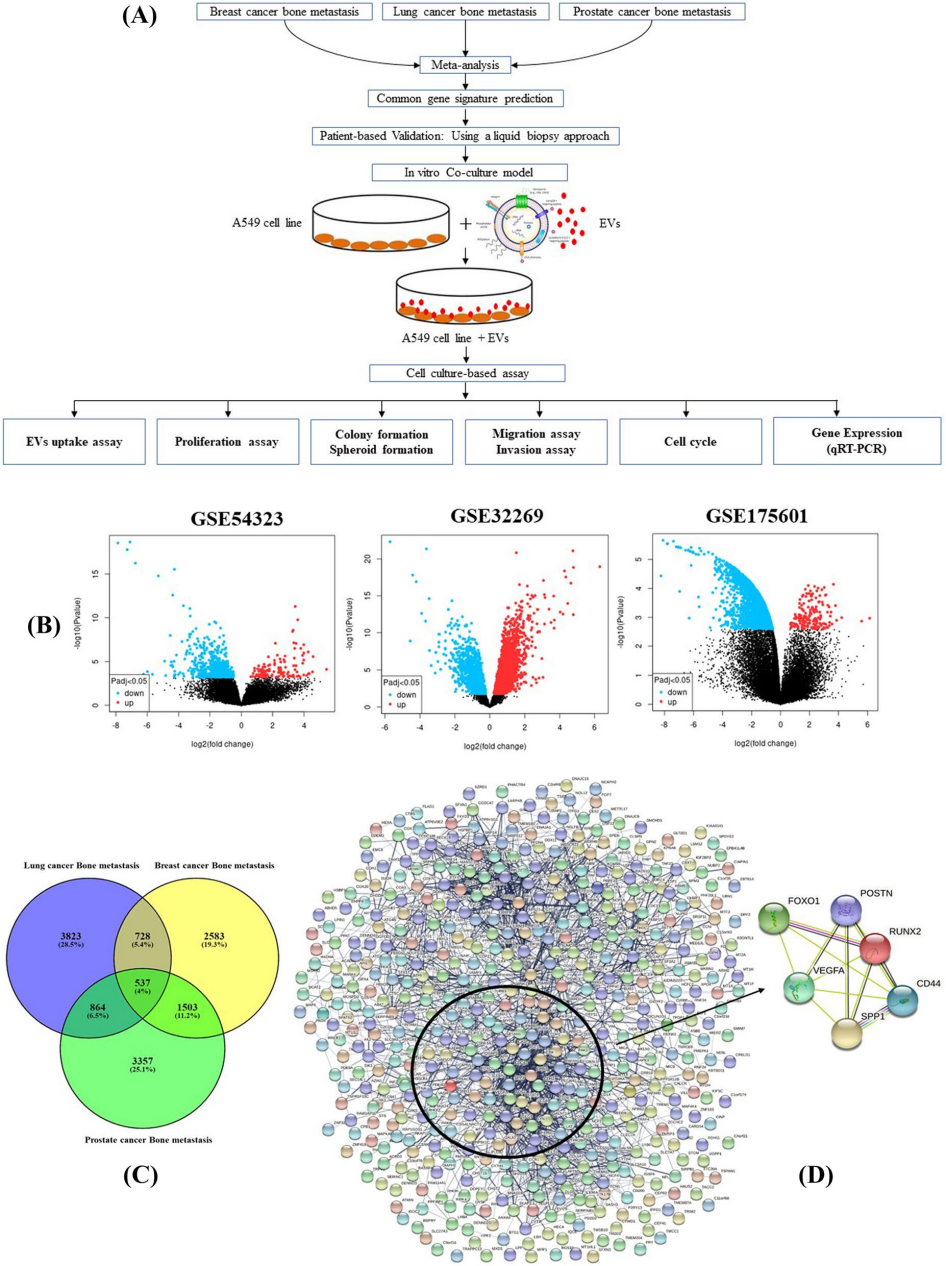
(**A**) The entire study workflow. (**B**) Volcanic plot of DEGs for four datasets: GSE54323, GSE32259, and GSE 175,601. Each dot represents an individual gene (Red dots presented upregulated genes with logFC > 2 and adj p value < 0.05, while blue dots presented downregulated genes with logFC < 1 and adj *p* value < 0.05). (**C**) Venn diagram representing the overlapping genes. Each of the circles represents a dataset of bone metastases with various primary tumors breast cancer, prostate cancer and lung cancer. (**D**) String protein interaction analysis. String output showing interaction of the common six genes (*SPP1*, *VEGFA*, *FOXO1*, *RUNX2*, *CD44* and *POSTN*) that are specific for bone metastases.

The entire study workflow is presented in Fig. 1A. Three datasets of gene expression arrays were obtained from the GEO database as follows: GSE54323, GSE32269, and GSE175601, respectively (Table 1). To identify the transcriptional signature between primary cancer and bone metastasis, the datasets were analyzed by GEO2R. By comparing expression profiles from primary tumors and tumors with bone metastasis in GSE54323, GSE32269, and GSE175601 datasets, 2583, 3823, and 3357 genes were identified as DEGs respectively. The volcano plot of the DEGs depending on FCs (FC 2, FDR 0.05) is shown in Fig. 1B. Finally, the 537 commonly expressed DEGs, including 294 upregulated and 243 downregulated genes were identified in the lung cancer bone metastasis compared with the lung cancer samples via the Venn diagram software in the three datasets (Fig. 1C).

**Table 1:** Information on the Selected GEO Datasets.

Series	Platform	Gene chip
GSE54323	GPL570	Affymetrix human genome U133 plus 2.0 array
GSE32269	GPL96	Affymetrix human genome U133A array
GSE175601	GPL21185	Agilent-072363 SurePrint G3 human GE v3 8 × 60 K microarray

In order to achieve essential candidate gene and vital gene modules in bone metastasis, PPI network analysis was performed. We perform the PPI network of the 537 overlapping candidate genes using the STRING biological database (Fig. 1D). The main communication network consisted of two main interconnected groups with CD44 and RPS15 as the hub nodes. Based on the strong interaction in the PPI network and the transcriptional profile, we selected six significant genes *SPP1, VEGFA, POSTN, RUNX2, CD44,* and *FOXO1.* Additionally, we validated these six genes using qRT-PCR in patient samples to establish their role in bone metastasis.

**Figure 2:**
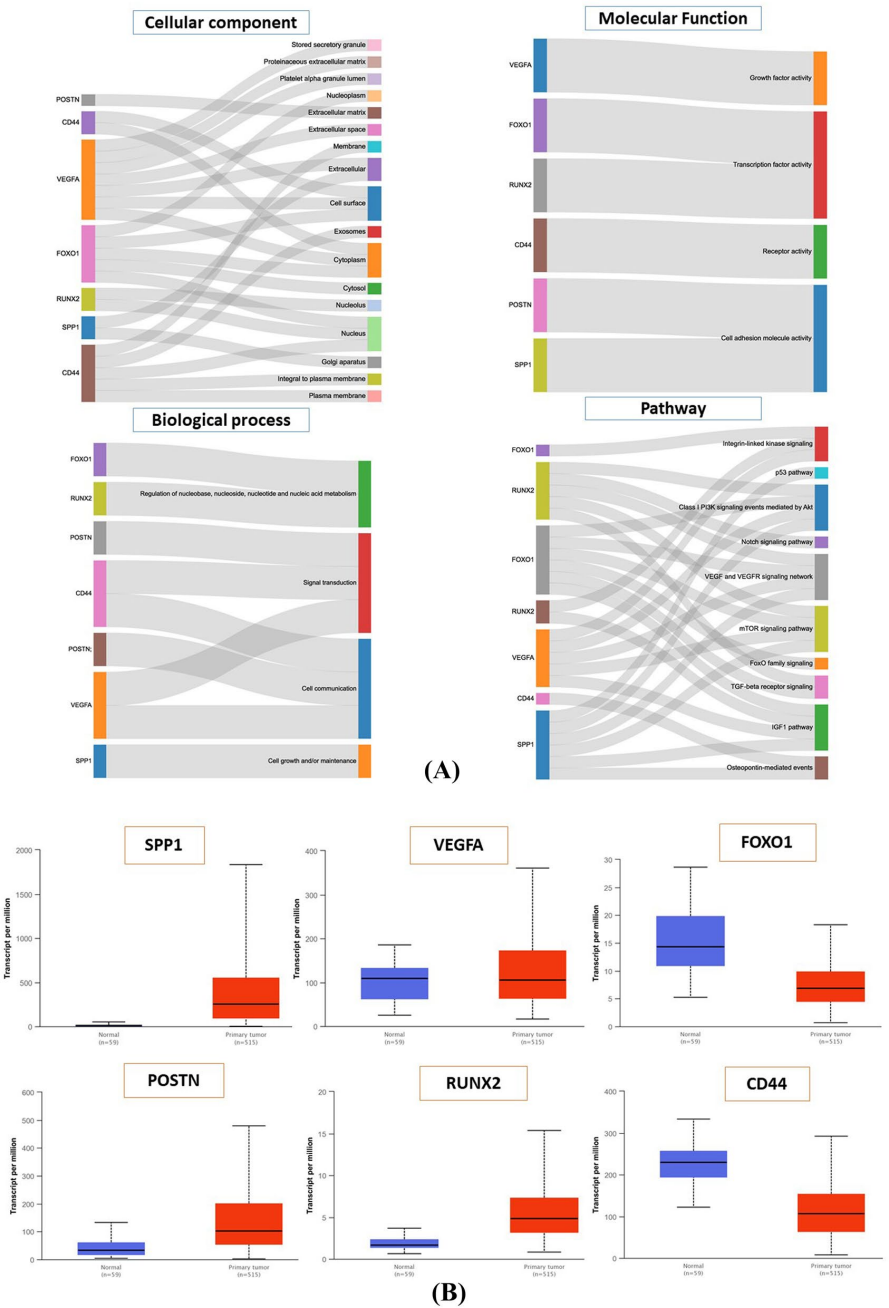

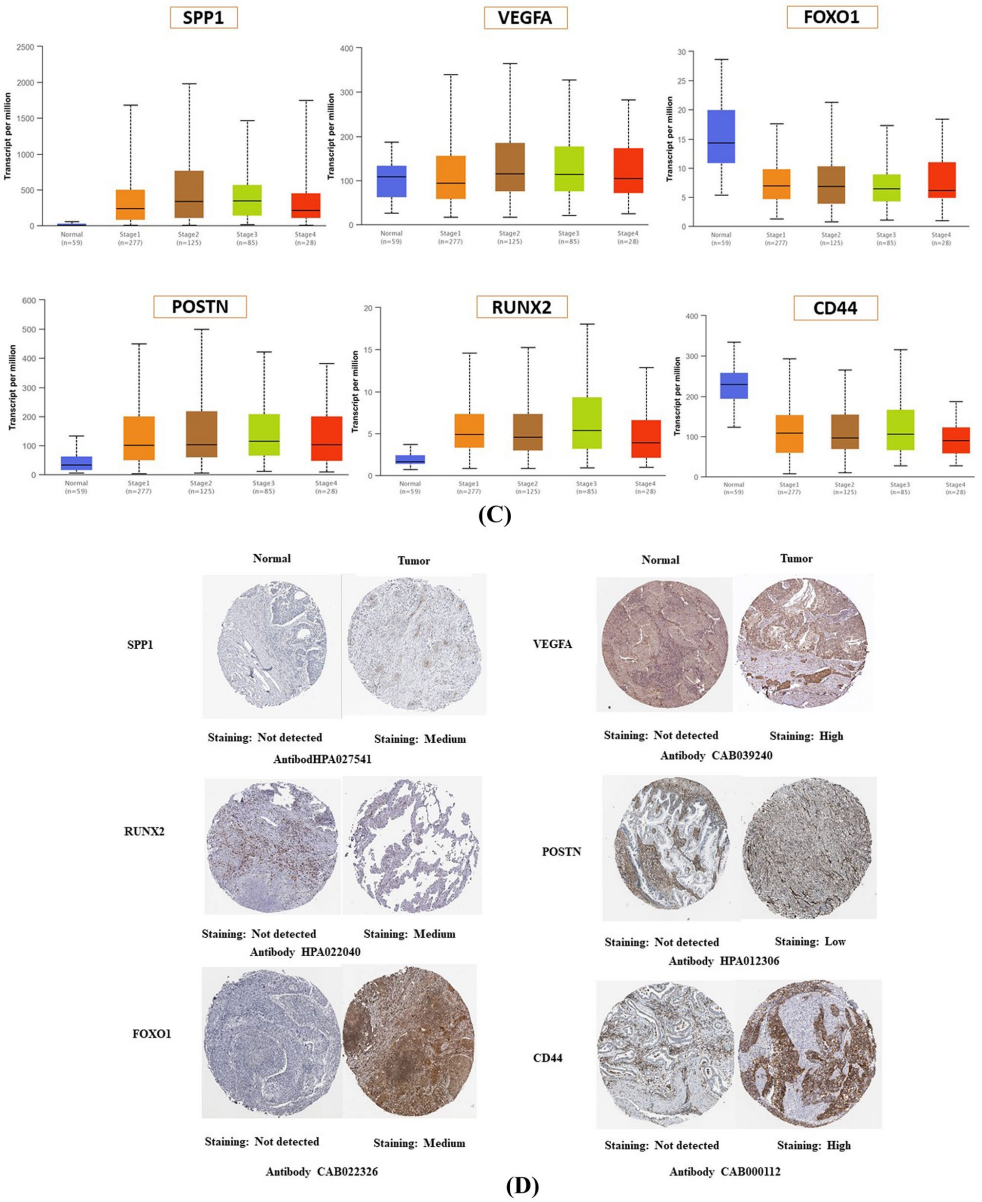
(**A**) The top six differentially expressed genes GO terms. Each of the graph show a biological process, molecular functions, cellular component and pathway involvement of the genes. (**B**) mRNA expression of *SPP1*, *VEGFA*, *FOXO1*, *RUNX2*, *CD44* and *POSTN* genes in lung cancer tissues and adjacent normal lung tissues. (**C**) Relationship between mRNA expression of hub genes and individual cancer stages of lung cancer patients. (**D**) Immunohistochemistry images of six genes in Lung cancer tissues and normal lung tissues (Human Protein Atlas).

To understand the DEGs functional levels, the GO analysis was carried out utilizing the online biology tool FunRich with a significant threshold of *p *< 0.05. The results of the six DEGs in the GO terms of the categories were divided into three groups as follows: biological process (BP), cellular component (CC), and molecular function (MF). GO term analysis showed that these genes were enriched for cell communication and cell adhesion (BP), nucleus and exosomes (CC), and cytokine and receptor activity (MF), respectively (Fig. 2A).

Furthermore, to discriminate the potential pathway of DEGs, we used KEGG pathway analysis. The analysis of the KEGG pathway revealed 20 significantly enriched pathways. The five most enriched pathways were: the VEGF signaling pathway, the TGFβsignaling pathway, the FOXO signaling pathway, PI3K-Akt signaling pathway, and the mTOR signaling pathway (Fig. 2A).

**Figure 3:**
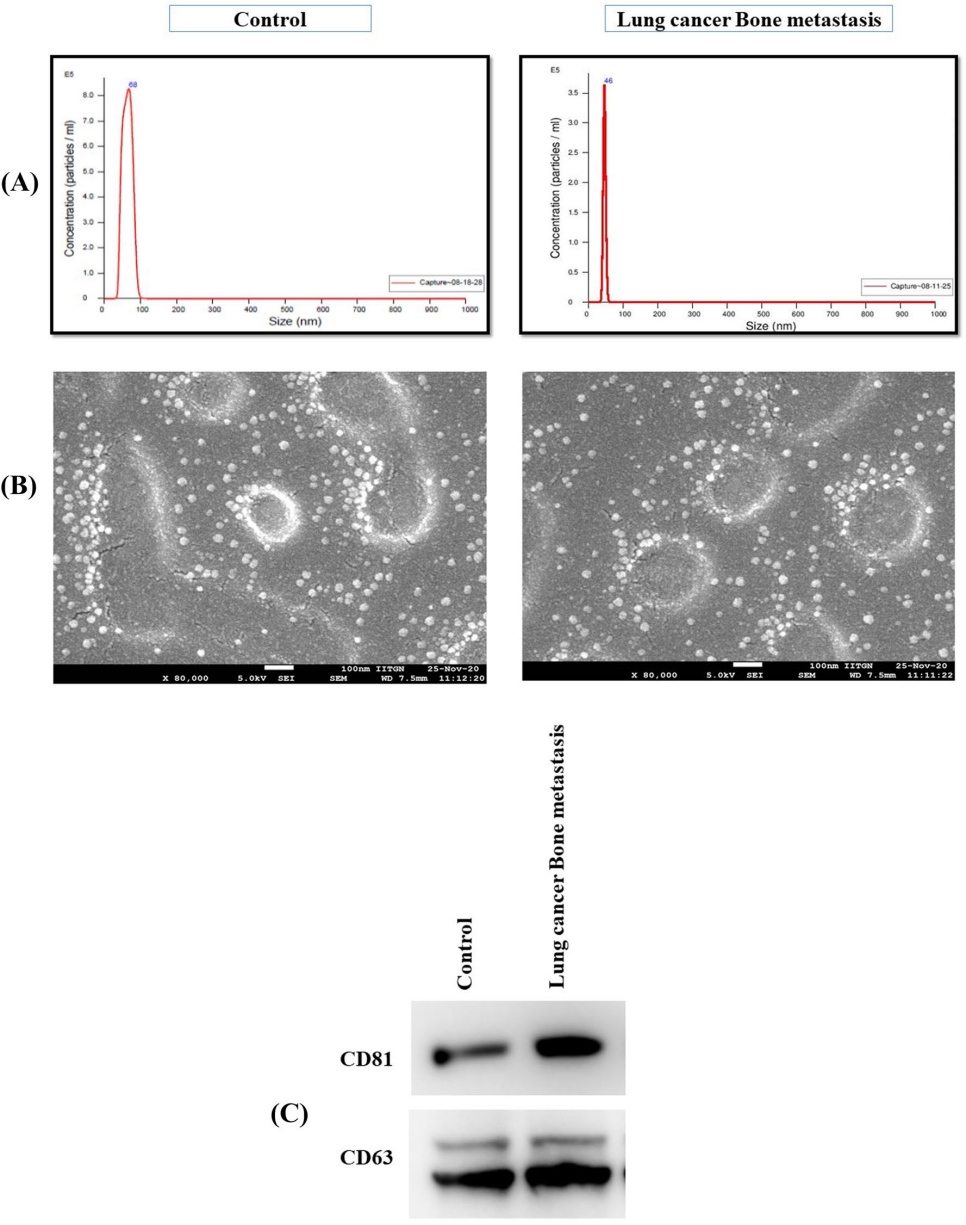
Characterization of extracellular vesicles using NTA, AFM and Westernblot. (**A**) Assessment of size and concentration of serum EVs from health person and lung cancer bone metastasis patients using Nanoparticle Tracking Analysis (NTA). The x-axis indicates the size distribution of particles and the y-axis shows the relative concentration (particles/ml) in NTA; (**B**) Representative AFM images of EVs from healthy person and lung cancer bone metastasis patient sera (Scale: 100 nm); (**C**) Western blot analysis of EVs isolated from serum of healthy individual and lung cancer bone metastasis patients to confirm the presence of classical common exosome markers (CD63 and CD81).

UALCAN is used to analyze the correlation between transcript levels and tumor stage in patients with lung cancer. Differential gene expression analysis of the six genes revealed significant upregulation of *SPP1, VEGFA, POSTN*, and *RUNX2* (p < 0.001), whereas *CD44* and *FOXO1* were significantly down-regulated in primary lung cancer patient samples compared to healthy control samples (p < 0.001) (Fig. 2B). The relationships between mRNA expression of the hub genes and clinical parameters in lung cancer patients were analyzed by UALCAN, such as individual cancer stages. As shown in Fig. 3C, mRNA expression of *SPP1, VEGFA, POSTN, CD44, FOXO1,* and *RUNX2* genes was significantly change with the cancer stages of the patients. According to the findings, patients with advanced cancer stages had either the lowest or highest level of mRNA expression for the majority of hub genes. The lowest expression of *SPP1* and *CD44* mRNA was found in stage 4, while the highest expression of *VEGFA* and *POSTN* mRNA was found in stage 4 (Fig. 2C).

The Human Protein Atlas database further confirmed these findings revealing low protein expression in normal lung tissues, and medium to high protein expression in advanced lung cancer tissues (Fig. 2D).

### Characterization of serum EVs derived from lung cancer with and without bone metastasis patients

First, we isolated extracellular vesicles from patients serum diagnosed with lung cancer with and without bone metastasis and healthy individual. These EVs were characterized by NTA, AFM, and western blot. The NTA results showed a single peak for the concentration of EVs in the size range of 40–50 nm (Fig. 3A). Serum EVs derived from lung cancer bone metastasis patients had a mean size of 45.8 ± 0.0 nm and a concentration of 5.15 × 10^7^ particles/ml. The control EVs had an average particle size of 68 ± 4.2 nm and a concentration of 3.12 × 10^6^ particles/ml. The EVs isolated from lung cancer bone metastasis patients contained a higher number of EVs and were smaller in size compared to the control. The AFM result indicated that sphere-shaped vesicles with a mean radius of 50–60 nm, which was consistent with the NTA profiles (Fig. 3B). In addition, EVs representative markers, including CD63, and CD81, were specifically enriched in these isolated EVs (Fig. 3C). These results confirmed that EVs were successfully isolated, most of which were exosomes.

### Differential gene expression analysis of EVs: identifying a marker for liquid biopsy

**Figure 4:**
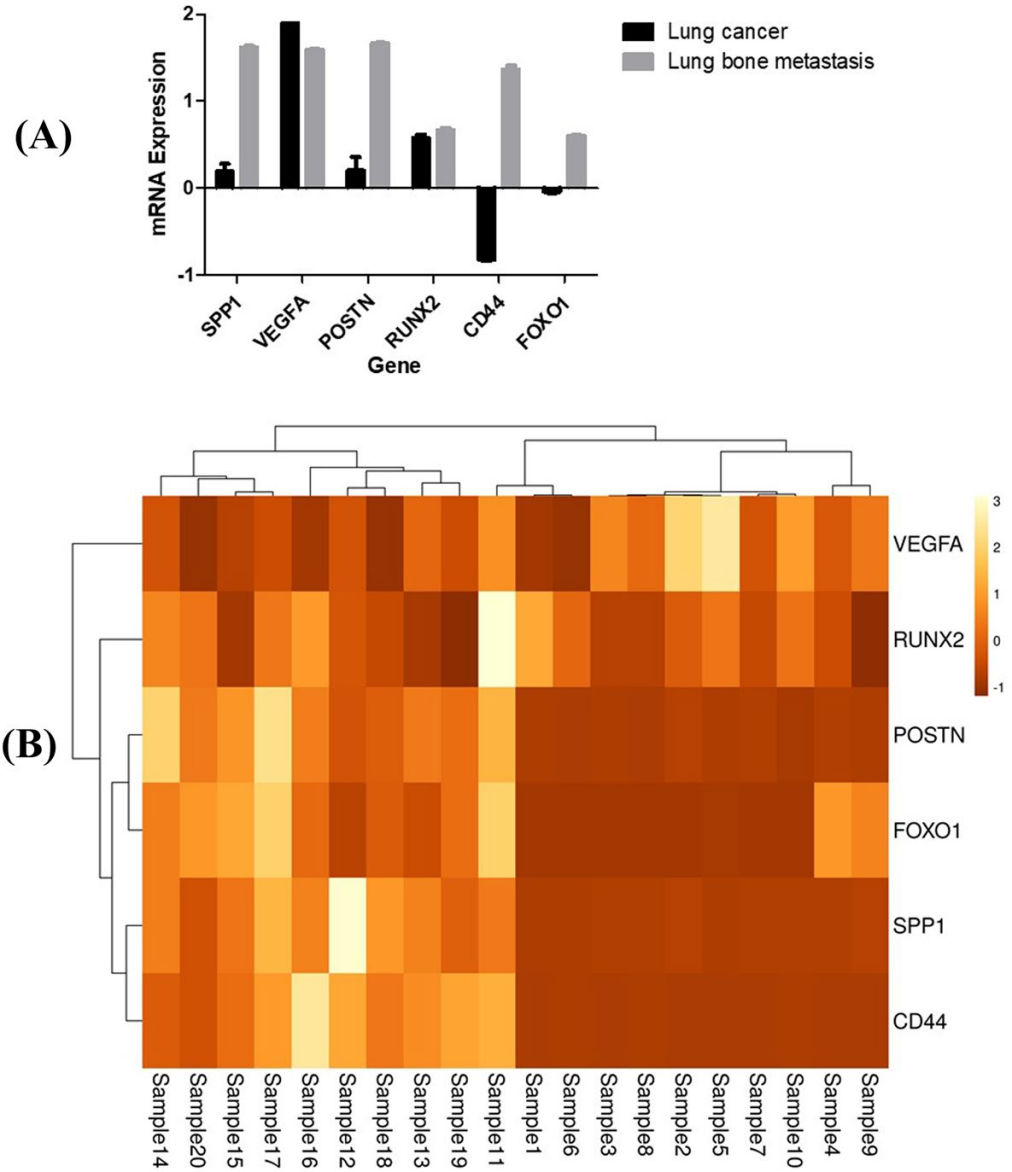
(**A**) Gene expression study using liquid biopsy approach. Primary lung EVs mRNA, advance stage lung cancer with bone metastases, as well normal EVs mRNA analysed with qRT-PCR. (**B**) Unsupervised hierarchical clustering of primary lung cancer with advanced stage bone metastatic samples. Clustering was based on six differentially expressed genes at a false discovery ratio level of 0.05. The colored bar specifies the variation in gene expression in target samples as compared to reference cells i.e., Brown, more expressed and cream, less expressed in target samples. Further, the black lines of the dendogram stand for the support for each clustering. The metric performed was Euclidean distance, with complete linkage for distance between clusters. 1 to 10 sample primary lung cancer and 11 to 20 samples for lung cancer bone metastasis.

To validate the results of DEGs for our meta-analysis that can discriminate metastatic cancer from primary cancer, we collected 10 healthy, 10 primary lung cancer, and 10 bone metastasis patients’ blood samples. Quantitative gene expression of *SPP1, VEGFA, POSTN, RUNX2, CD44,* and *FOXO1* from lung cancer patients with and without bone metastasis normalized against healthy control. Lung cancer patient-derived EVs mRNA showed upregulation of *SPP1, VEGFA*, and *POSTN* as compared to lung cancer with bone metastasis patients. Interestingly, *CD44* and *FOXO1* transcripts were observed to be down-regulated in primary lung cancer but significantly up-regulated in lung cancer patients with bone metastasis. However, no significant change was observed in the expression of the *RUNX2* gene (Fig. 4A).

In addition, we also performed the hierarchical cluster to check whether the selected six genes would be important in categorizing primary lung cancer into sets that have a variety of possible development of bone metastasis or not. The expression profile of the six genes was therefore used in hierarchical clustering to classify a group of 10 non-metastatic lung tumors as depicted in Fig. 4B. The lung cancer was clustered into two individual groups, based on their gene expression profile and correlation in primary and bone metastatic. We predicted that tumors with a higher fold change in gene expression profile would have a poorer prognosis, which could be caused by disease progression to bone metastasis.

### Internalization of EVs by A549 cells

**Figure 5:**
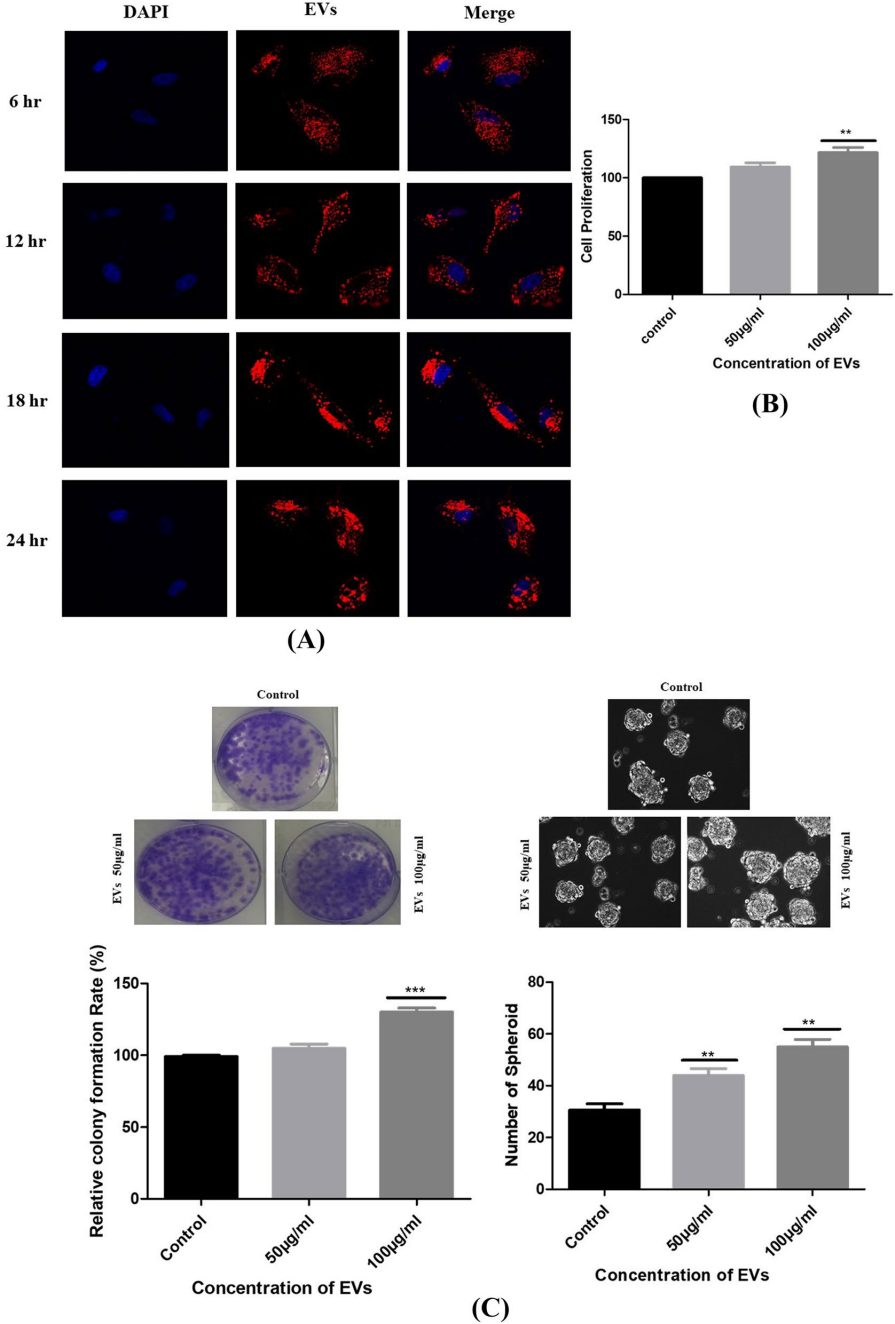

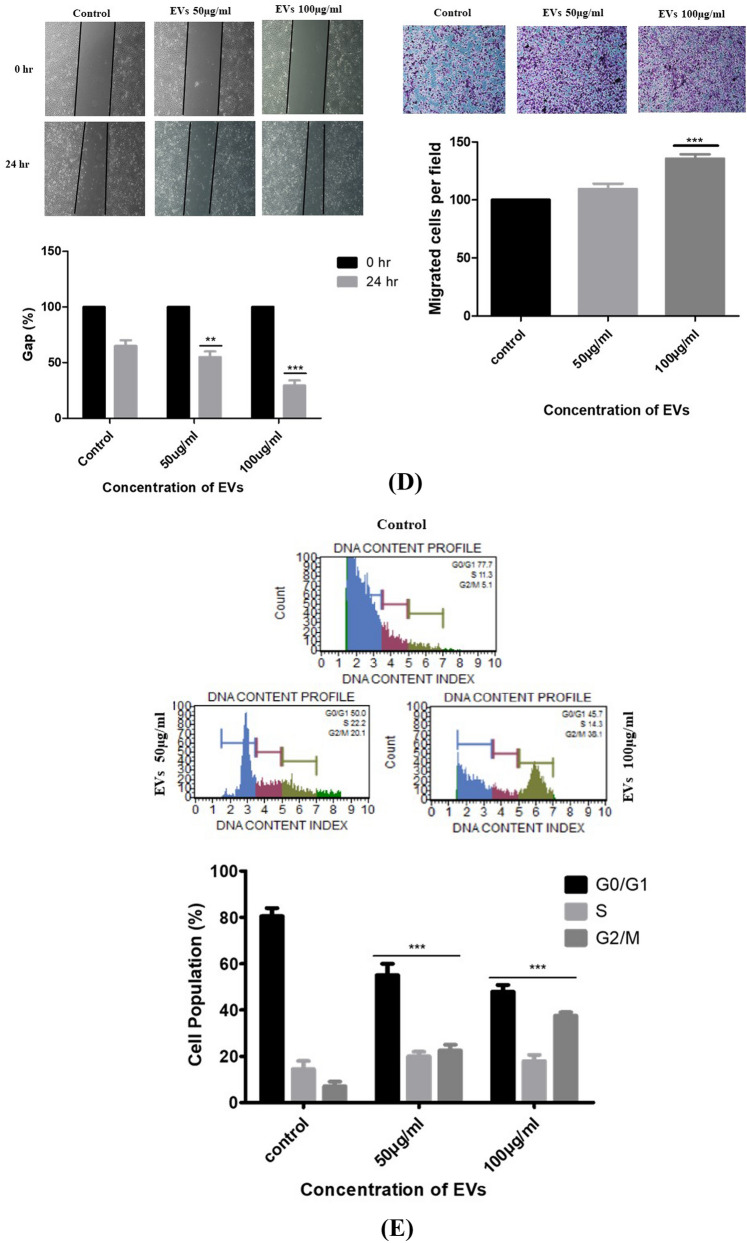

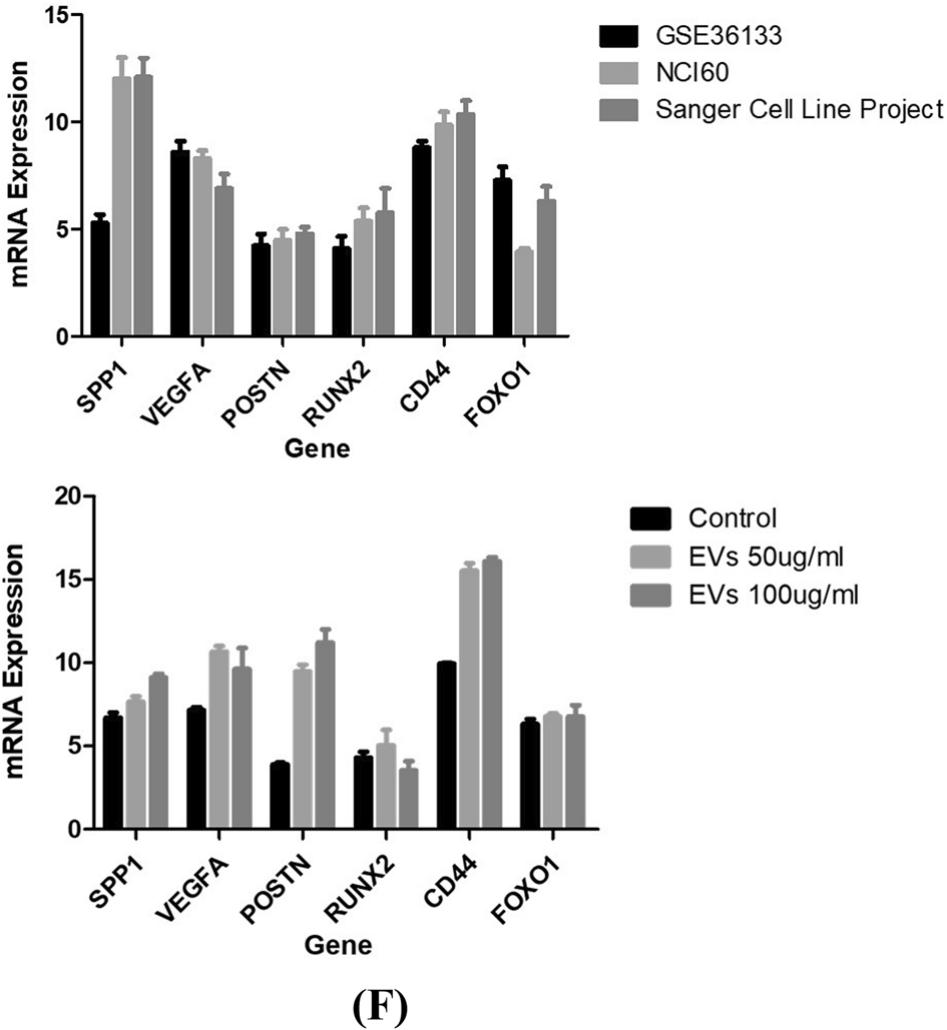
(**A**) Internalized lung cancer bone metastasis derived EVs induce malignant transformation of the recipient A549 cells. Fluorescent images of EVs uptake by A549 cells. EVs were stained with DIL dye, and then coculture with A549 for 6 h, 12 h, 18 h and 24 h. Nuclei were stained with DAPI. (**B**) Cell proliferation assay was assessed for different concentration (50 and 100 µg/ml) for 24 h using MTT assay. MTT results showed a significantly increased cell proliferation of EVs-treated cells. Data represented as mean ± SEM of three independent experiments. (**C**) Colony and Sphere forming ability of A549 cells was analyzed post EVs treatment. Results indicated that the significantly increased cell colony and sphere forming ability of EVs-treated cells. Data represented as mean ± SEM of three independent experiments. (**D**) The migration and invasion ability of A549 cells was monitored in EVs treated condition by wound healing assay and transwell assay. The graphs represent the relative distance migrated by cells per field (μm) in the wound healing assay and migrated cells from the transwell. Error bars represent mean ± SEM of three independent experiments with *p*-value. (**E**) The effect of EVs on cell cycle mechanism was assessed using MuseTM analyser based flow cytometry analysis. The graph shows the percentage of cells in each phase of the cell cycle. Error bars represent mean ± SEM of three independent experiments with *p*-value. (**F**) RT-PCR validation of potential markers in EVs with and without treated A549 cells. Relative gene expression profile of six marker panel in treated and control cells after 24 h. The expression of mRNAs increased after EVs exposure, but they showed different kinetics. Data shown as mean ± SD with statistical significance.

EVs secretion is a vital approach for tumors to induce systemic changes, whereas how they affect and control the behavior of recipient cells is still unclear^28^. Thus, we examined the effect of EVs on biological processes (e.g., proliferation, migration, colony formation, etc.) of A549, which is considered classical in vitro models^29^. First, we assessed whether healthy and lung cancer bone metastasis patients derived EVs were internalized by A549 cells. High-throughput microscopy showed that A549 cells indeed took up EVs (labeled red by the Dil dye) as early as 6 h after treatment. Prominently, after 24 h, the majority of A549 cells were loaded by EVs (Fig. 5A). Indeed, image analysis revealed a 91% internalization efficacy. Furthermore, the results indicated that EVs derived from lung cancer bone metastatic serum were localized around the nucleus and were time-dependent. Surprisingly, when compared with healthy control EVs, slight uptake was observed, demonstrating that healthy person-derived EVs have no significant role in A549 cells (Supplemental Fig. S1). This suggests that functional alterations demonstrated by this study were due to EVs-induced cell population, and not individual cell-level effects.

### Functional impact of EVs on A549 cell proliferation

Tumor cells can release EVs cargo into the microenvironment. Since EVs can willingly be absorbed by recipient cells, so we questioned whether oncogenic phenotypes might be transmitted from more aggressive cells to idle poorly metastatic cells^30^. Then, we determined whether the internalized EVs could induce bone metastasis like the malignant transformation of the A549 cells. To assess the functional impact of patient-derived EVs on A549 cells, we co-cultured A549 cells with 50–100 μg/ml of EVs, in a DMEM medium containing 10% exosome-depleted FBS, for 24 h. In the control set, cells were not treated with EVs. Cell number was compared between the EVs treated and the control group. By using the MTT assay, we found that the proliferation rate of the A549 cells significantly accelerated 24 h after EVs exposure suggesting that EVs can promote cell proliferation (Fig. 5B).

Since the EVs were isolated from lung cancer bone metastasis patients, we were then fascinated to uncover whether the above alterations also resulted in the de novo appearance of metastasis features in the transformed A549 cells. To answer this question, we investigated the effect of patient-derived EVs on colony and spheroid formation. Our results showed a higher number of colonies and spheroids in the EVs-treated group compared to the control. These findings are suggestive of the potential role of EVs on cancer cell growth and progression (Fig. 5C).

### EVs promoted A549 cell migration and invasion in vitro

EVs secretion is a crucial mode for tumors to prompt migration and invasion, whereas how they promote cell migration is still unclear. To investigate the effect of EVs derived from lung cancer bone metastasis patients on the migration potential of A549 cells, we used a 2D scratch assay. The results of the scratch closure test show that the migration of A549 cells increased significantly after 24 h in the presence of EVs compared to the control group (*p* < 0.001) (Fig. 5D). Further, a dose response was noted with the 100 μg/ml dose indicating the greatest migration rate compared to the 50 μg/ml. Similarly, a transwell migration assay detected that EVs could promote A549 migration in a dose-dependent manner (Fig. 5D). Taken together, these results specified that EVs treatment derived from lung cancer bone metastasis patients could upregulate the migration ability of A549 cells.

### Effects of EVs on cell cycle

The cocultured A549 cells with EVs were also subjected to cell cycle progression using MUSE green laser portable flow cytometer. The data showed that treatment with 50 µg/ml of EVs shows a low proportion of cells in a G0/G1 phase but the higher proportion in the S phase compared to the control. Furthermore, treatment with 100 µg/ml of EVs showed an increased proportion of cells in the G2/M phase compared to the control. There were significant differences in the cell cycle phase between the EVs‐treated and control groups at 24 h. The results indicate that the EVs derived from metastatic patients play a significant role in cell cycle progression (*p* < 0.05) (Fig. 5E). Overall, these in vitro cell-based assays suggest a possible role of EVs in cell proliferation, migration, and invasion with a propensity for G2/M transition.

### Tumor‐derived EVs influence the gene expression of A549 cells

First, we identified the expression data of six genes in the lung cancer cell line A549, which were reported using microarray, the data of which were deposited in the CellExpressed system. The gene expression levels of *SPP1, VEGFA, POSTN, RUNX2, CD44,* and *FOXO1* were studied in A549 cell lines and results showed that the expression of the respective gene was high in the A549 cell line (Fig. 5F).

Next, we assessed whether the above effects of EVs inducing bone metastasis like the transformation of the A549 cells were accompanied by a cellular-molecular oncogenic reprogramming of the target cells. Quantitative gene expression patterns of *SPP1, VEGFA, POSTN, RUNX2, CD44,* and *FOXO1* were analyzed from the in-vitro model generated after A549 cells co-incubation with EVs exposure at 50 and 100 µg/ml concentration for 24 h. A549 cells without EVs treatment were used as control and the results were compared. It was shown that the expression of *SPP1, CD44*, and *POSTN* genes was upregulated in a dose-dependent manner as compared to the control. Additionally, *VEGFA* showed an increase in expression at 50 µg/ml concentration but downregulated at 100 µg/ml concentration as compared to the control. However, no significant changes were observed in the expression level of *RUNX2* and *FOXO1* compared to the control (Fig. 5F). The results have shown that lung cancer bone metastasis-derived EVs could be taken up by cells and change their gene expression and phenotype, subsequently in cell affinity to halt the cell cycle and accelerate migration.

## Discussion

Given those metastatic consequences account for about 90% of cancer-related mortality^31,32^, thorough knowledge of tumor metastatic processes is one of the most critical issues in both fundamental and applied tumor biology research. While Paget's "seed-and-soil" hypothesis addressed some organotropic properties of tumor cells in the early nineteenth century^33^, an extended model is needed to accurately describe the spatiotemporal characteristics of metastatic processes. As a result, many studies over the past ten years have revealed that the soluble and vesicle-like elements of the tumor microenvironment are critical for the formation of metastatic spread^34^. Recent research on the mechanisms behind intercellular communication has undoubtedly underlined the critical role that EVs, such as exosomes, play in acting as "information bundles" for coordinated cell-to-cell signaling^35^. However, fundamental molecular and cellular mechanisms have not yet been explored. Nevertheless, the trafficking procedures that manage the discharge of extracellular vesicles from tumor cells and uptake by the target recipient cells remain largely unknown. The present study elucidated for the first time the role of patient-derived bone metastasis EVs in the A549 cell line regarding proliferation, migration, invasion, and cell cycle activation with differential expression of some key genes involved in key regulatory pathways using an in vitro coculture model.

Current scenario meta-analysis methodologies have been used to identify clinically significant biomarkers in various cancers, for example, osteosarcoma, prostate, and pancreatic cancer^36–38^. In the present study, we performed a comprehensive meta-analysis of microarray gene expression datasets derived from patients with and without bone metastasis to identify a panel of genes that are commonly found bone metastasis. The primary objective was to identify genes that are consistently overexpressed in all bone metastasis patients regardless of the primary site of cancer. We found six genes such as *SPP1, VEGFA, POSTN, RUNX2, CD44,* and *FOXO1* that were consistently overexpressed in patients with bone metastasis compared to non-metastatic tumors. Since EVs are routinely shaded from primary tumors and entered circulation, we speculated if we could detect any of these six shortlisted genes in an actual patient’s blood sample, then we can eventually establish a non-invasive biomarker for early prediction of bone metastasis. Thus, we validated this identified gene in patient serum derived EVs. In response to that, we isolated EVs from sera of healthy, primary lung cancer and lung cancer with bone metastasis patients. Initially, we wanted to carefully characterize the EV population that we would use for our validation of gene panel and in vitro experiments. We adopted the experimental guidelines of the International Society for Extracellular Vesicles (ISEV), which include the determination of form, size distribution, and protein markers^39^. The isolated EVs were confirmed by positive expression of three CD antigens (CD63, CD81, and CD9) by western blotting as previously published literature^40^. Further, the size and shape of EVs were confirmed by AFM and NTA. Based on transcript level expression study using quantitative real-time PCR showed five significantly differentially expressed genes *SPP1, VEGFA, CD44, FOXO1* and *POSTN* in EVs cargo derived from lung cancer patients with bone metastasis. This showed possibility of differential packaging of tumor-derived transcription during exosome biogenesis^41^. Future studies in a large set of patients are needed to validate the clinical value of the gene expression panel as a predictive biomarker for bone metastasis in patients with lung cancer. Increasing evidence suggests that EVs are key mediators of tumor metastasis and early predictors of tumor invasion and progression^42^. Taken together, our findings demonstrate that the EVs-based gene expression study can be used for monitoring lung cancer patients for bone metastasis for better clinical outcomes after validation on a larger set of patients. This prompted us to explore the role of EVs in the modulation of target niche behavior using a coculture in-vitro model.

The human lung carcinoma cell line A549 was chosen for this in vitro coculture model development to assess EVs uptake and its impact on cell proliferation, migration, invasion & cell cycle. Given the appropriate uptake of these vesicles by the targeted A549 cells, it is highly probable that most of the gene expression mentioned above was significantly affected. According to previous results in the literature, we showed successful internalization of EVs by recipient A549 cells. Peindo and their team suggested that the systemic delivery of fluorescently labeled exosomes from melanoma cells results in the dissemination of exosomes to both lung and bone tissue, and metastatic development co-localized with exosome deposition sites^43^. As expected, after 24 h of exposure, A549 cells showed accelerated proliferation probably initiated by the transforming EV cargo. Li et al. recently reported that exosomes released from PLC/PRF/5 promoted cell growth by inducing proliferation in MHCC-97H cell lines^44^. This proliferation strongly suggested that EVs initialize and sustain the malignant transformation of A549 cells.

To understand the mechanism of cell proliferation, it was imperative to assess the effect of EVs on colony/spheroid formation, migration, invasion, and cell cycle progression. Cell motility and anchorage-independent colony formation are the main factors responsible for cancer progression. Patient-derived EVs induced morphological differences and a significant increase in colony/sphere formation (Fig. 5C). Additionally, it is suggested that a very small population of cancer cells that transfer the appearances of somatic stem cells and can develop into diverse cell forms within cancer cells. These CSCs are capable of self-renewal, differentiation, and metastatic dissemination and are resistant to chemotherapy/radiation therapy. Interestingly, the CSC marker CD44 also increases after the treatment of EVs. These observations led us to propose that the EVs cargo contains specific ligands (gene products) that can stimulate oncogenic pathways in target cells and ultimately lead to metastasis. In addition to this we also provide evidence that EVs derived from lung cancer bone metastasis could prompt migratory and invasive abilities of A549 cells by the transfer of molecules through EVs. Recent studies have also suggested that the exosome promotes migration in hepatocellular carcinoma, prostate cancer, and pancreatic cancer^45–47^. To corroborate these findings we performed cell cycle analysis and showed that EVs treatment has a notable effect on G0/G1 and G2/M phase transition suggesting that the role of EVs on cell proliferation may be due to cell cycle dysregulation. Thus, bone metastasis derived EVs could modify A549 cells in their environment to facilitate tumor growth, invasion and metastasis.

In addition to revealing tumor markers, exposed cells undergo significant changes in their gene expression patterns. As was previously mentioned, particular gene expression changes are frequently linked to particular stages of the development of cancer. Importantly, we successfully identified three metastasis-specific markers, *SPP1, CD44*, and *POSTN* in the EVs-exposed A549 cells. The presence of these markers in EVs-exposed A549 cells shows effective transfer of the encoded tumorigenic information and may play a pivotal role in disease progression. One of the earlier studies reported that *SPP1* can stimulate *VEGF* growth through an autocrine and paracrine mechanism leading to tumorigenesis and metastasis^48^. Furthermore, *SPP1* also binds to *CD44,* causing cell signaling that mediates tumor progression and metastasis^49^. Additionally, the study by Wang, YY et al. also reported that *CD44* stimulates the migration and invasion capacities of lung cancer cells through ERK–ZEB1 signaling^50^. Our present study also corroborated these previous reports, suggesting that serum *POSTN* was a self-governing survival biomarker in patients with lung adenocarcinoma patients with bone metastasis^51^. Furthermore, Che et al. demonstrated that down-regulation of Periostin expression modulated the tumor microenvironment and inhibited bone metastasis by blocking the integrin signaling pathway in lung cancer^52^. Therefore, these three genes can be the main predictors of bone metastasis independent of regular prognostic conditions and may potentially be useful for routine patient staging.

Finally, it is crucial to emphasize that our conclusions are not solely based on in silico predictions, but also on meticulously planned and methodically carried out in vitro experiments, all of which demonstrate that the distinct, robust molecular content of isolated EVs can create a special intercellular niche responsible for the re-education of nearby cells via neoplastic transformation. Besides, the present study provided the first evidence that EVs derived from a lung cancer patient with bone metastasis may play a crucial role in the development of the pre-metastatic niche. So, the old "seed-and-soil" paradigm must be supplemented and extended with the oncosome-driven re-education process, according to our findings and newly published further EVs evidence.

## Material and methods

### Integrative meta-analysis

An electronic database search was performed using the MEDLINE/ PubMed resources to retrieve various relevant articles related to microarray studies involving bone metastasis in different primary cancers. Gene expression datasets GSE54323, GSE32269, and GSE175601 were obtained from the Gene Expression Omnibus (GEO) database (https://www.ncbi.nlm.nih.gov/geo/), which included raw counts of RNAseq expression data and corresponding clinical information.^18^. The details of the datasets are provided in Table 1. We used the GEO2R online tool to analyze the raw data of microarrays and identify DEGs between the primary tumor and metastasis tumor. GEO2R (http://www.ncbi.nlm.nih.gov/geo/geo2r/) is an interactive web tool that allows users to compare different groups of samples in a GEO series to examine differentially expressed genes according to experimental conditions. | log FC|> 2 and P < 0.05 were used as the cut-off standards to obtain DEGs. Eventually, a Venn diagram was used to identify common gene signatures for bone metastasis.

In the current study, STRING (http://string-db.org; version 11.0) was used to describe protein co-regulation of shortlisted genes (respective proteins) and measure functional interactions among nodes. The interaction specificity score > 0.4 (the default threshold in the STRING database) was considered statistically significant. The FunRich database can provide systematic and integrative functional annotation tools for users to investigate the biological meaning behind the list of genes. Gene ontology (GO) analysis including biological process (BP), cellular component (CC), molecular function (MF), and Kyoto Encyclopedia of Genes and Genomes (KEGG) enrichment analysis were performed for the selected genes by FunRich and then visualized in the graph. *p* < 0.05 was considered statistically significant. The functional enrichment of BP, CC, MF, and KEGG was analyzed and plotted using SRplot (http://www.bioinformatics.com.cn/en).

### UALCAN

The expression of individual DEGs was further verified using the UALCAN tool (http://ualcan.path.uab.edu), which determines relative gene expression across normal and tumor samples in TCGA datasets of 31 cancer types by performing a sample t-test (p-value ≤ 0.05)^19^. We examine the relative fold change expression of *SPP1, VEGFA, POSTN, RUNX2, CD44,* and *FOXO1* in lung cancer and normal samples concerning clinical pathologic characteristics (eg, cancer stages).

### Human protein atlas

The Human Protein Atlas (https://www.proteinatlas.org/) is a database of immunohistochemistry (IHC) based protein expression profiles in diverse cancers, normal tissue as well as cell lines^20^. IHC images of the identified cohort *SPP1, VEGFA, POSTN, RUNX2, CD44,* and *FOXO1 *protein expression in clinical specimens of lung and normal tissues were obtained from this database.

### Study design and participant consent

This study was reviewed and approved by the Gujarat University ethics committee for human subject research, and all research was carried out according to guidelines, and written informed consent was obtained from all patients. Blood samples were collected from patients diagnosed with lung cancer and lung cancer bone metastasis from Vedanta hospital and Civil hospital, Ahmedabad. Blood samples from a healthy individual (matched for age and gender with the patients) with no etiological history of smoking were used as normal controls. Clinical-Pathological details include tumor location, histopathology, age, gender, habit, and the stage noted in each case. Patients with multiple metastases, HIV/HBsAg positive were excluded from the present study.

The validation of selected genes predicted from the meta-analysis was performed in EVs mRNA derived from serum samples using quantitative real-time PCR. 10 healthy persons, 10 primary lung cancer, and 10 lung cancer bone metastasis patients’ blood samples were collected with prior consent. Blood in containers without anticoagulant or coagulant was kept at room temperature for 30 min and then at 4 °C till separation (less than 4 h) to ensure serum separation. Serum samples were centrifuged at 5000 rpm for 10 min, and then at 3000 rpm for 10 min and stored at − 80 °C before use.

### EVs isolation

Blood samples from lung cancer, lung cancer bone metastasis patients, and healthy controls were centrifuged at 2000×*g* for 10 min at 37 °C to remove cells and debris. The supernatant was transferred to a new sterile tube, and the EVs were precipitated using a miRCURY exosome kit for serum/plasma as per the manufacturer’s protocol (Cat. No./ID: 76603). The EVs pellet was re-suspended in 100 μl of phosphate-buffered saline (PBS) and stored at − 80 °C until further analysis^21^.

### EVs characterization

The size distribution and concentration of EVs were measured using a Nanoparticle Tracking Analysis (NTA) on NanoSight NS300 (Malvern Instruments Ltd). Purified EVs were resuspended in PBS and injected into the sample chamber. Each sample was measured three times. All the parameters of the analysis were set at the same values for all samples and three 60-s videos were recorded in all cases. The background was measured by testing filtered PBS, which revealed no signal.

Further, the size of EVs was also determined using Atomic Force Microscopy (AFM). The EVs were sonicated for 30 min for homogenization. First, 3 µl of purified EVs were fixed with 100 µl of 2% paraformaldehyde; then, a small drop of the solution was placed on a slide. The sample was subjected to air drying for 15 min before proceeding to AFM (JPK-Nano Wizard II) analysis^22^.

Additionally, the exosomal markers were also detected by western blotting. The EVs samples were re-suspended in 4 × sample buffer (NuPAGE LDS Sample Buffer (4X), NuPAGE Sample Reducing Agent (10 ×), Thermo Fisher Scientific), boiled at 96 °C for 7 min, and immediately cooled on ice. Electrophoresis of the proteins was performed using 4–12% Bis–Tris Protein Gels (NuPAGE Novex, Thermo Fisher Scientific), at 200 V and 0.03 A for 40 min. The proteins of electrophoresed gels were transferred to the PVDF transfer membrane (Millipore, Darmstadt, Germany) using transfer buffer [NuPAGE Transfer Buffer (20 ×)] at 30 V and 170–110 mA for 60 min. Membranes were blocked in EveryBlot Blocking Buffer (Bio-Rad) for 60 min at room temperature (RT). After blocking, membranes were incubated with each primary antibody overnight at 4 °C. Membranes were washed three times for 10 min with TBST buffer and incubated for 60 min at room temperature with secondary antibody in TBST buffer containing 1% nonfat milk. Membranes were washed three times for 10 min with TBST buffer. For EVs marker identification western blot analyses were performed with an anti-CD63 polyclonal antibody (1:250 dilution; Biorbyt, Cambridge, UK), anti-CD81 monoclonal antibody (clone: EAT2, 1:1000 dilution, LifeSpan Biosciences, Seattle, WA), and anti-rabbit IgG HRP-Conjugated antibody (1:1,000 dilution, R&D Systems). Bound antibodies were visualized by chemiluminescence using an ECL plus western blotting detection system (Advansta, Menlo Park, CA). We have submitted all relevant data of our experiments to the EV-TRACK knowledgebase (EV-TRACK ID: EV220330)^23^.

### Patient-based validation: gene expression analysis by qRT-PCR

Total RNA from EVs was isolated using TRIzol reagent (Invitrogen, Carlsbad, CA, USA) following the manufacturer’s protocol. EVs RNA was isolated from the primary and metastatic patients as well as healthy individuals. RNA integrity was further determined using a Bioanalyzer. The extracted RNA was stored at − 80 °C until further analysis. 1 μg total RNA was reverse transcribed to cDNA using the cDNA archive kit (Qiagen; Cat no: 205411) in 50 μl reaction volume following the manufacturer’s instructions and the resulting cDNA was used as a template for Real-Time PCR analysis. qRT-PCR was performed using QuantStudio 5 (Applied Biosystems, Foster City, CA, USA) with an SYBR Green PCR Master Mix. Primer sequences are provided in Table 2. Amplification was performed at the following cycling conditions: 1 cycle of 3 min at 95 °C for the initial denaturation step and 40 cycles of 5 s at 95 °C for the denaturation step, 20 s at 60 °C for the annealing and extension step. Melting curve analysis was performed following the amplification to differentiate the accumulation of specific reaction products from non-specific products or primer dimmers. The generation of a specific PCR product was tested using the ΔΔCT method with *β-actin* as the endogenous control. All experiments were performed in triplicates independently and the average C_T_ value was calculated for the quantification fold change analysis.

**Table 2:** Primer list.

S. no	Gene name	Sequence	No of base	Accession no
1	CD44	FP-5′-TCCAACACCTCCCAGTATGACA-3′	22	NM_000610
		RP-5′-GGCAGGTCTGTGACTGATGTACA-3′	23	
2	SPP1	FP-5'-CGAGGTGATAGTGTGGTTTATGG-3′	23	NM_000582
		RP-5'-GCACCATTCAACTCCTCGCTTTC-3′	23	
3	VEGFA	FP-5′-CTTGCCTTGCTGCTCTACC-3′	19	NM_003376
		RP-5′-CACACAGGATGGCTTGAAG-3′	19	
4	FOXO1	FP-5′- CTACGAGTGGATGGTCAAGAGC-3′	22	NM_002015
		RP-5′- CCAGTTCCTTCATTCTGCACACG-3′	23	
5	POSTN	FP-5′- CAGCAAACCACCTTCACGGATC-3′	22	NM_001135934
		RP-5′- TTAAGGAGGCGCTGAACCATGC-3′	22	
6	RUNX2	FP-5′-CCCAGTATGAGAGTAGGTGTCC-3′	22	NM_001015051
		RP-5′-GGGTAAGACTGGTCATAGGACC-3′	22	
7	β- ACTIN	FP-5′-TGACGTGGACATCCGCAAAG-3′	20	NM_001101
		RP-5′-CTGGAAGGTGGACAGCGAGG-3′	20	

### Cell lines and cell culture condition

The human lung adenocarcinoma alveolar basal epithelial A549 cell line was obtained from the National Center for Cell Science (NCCS), Pune, Maharashtra, India. A549 cells were cultured in Dulbecco's modified Eagle's medium (DMEM) medium (Gibco, Life Technologies, USA). The culture media were supplemented with 10% Gibco™ Exosome-Depleted fetal bovine serum (FBS) and PSN antibiotic solution (Gibco, Life Technologies, USA). Cells were kept in a humidified atmosphere of 5% CO_2_ at 37 °C. Exponentially growing cells were used throughout the study.

### EVs uptake assay

To examine the uptake of healthy and lung cancer bone metastasis derived EVs by A549 cells, 1 × 10^6^ cells were seeded on a 12-well plate (Corning, NY, USA) a day before the treatment. EVs were labeled with Dil dye (1,1′-dioctadecyl3,3,3′,3′-tetramethylindocarbocyanine perchlorate, PromoKine, Heidelberg, Germany) according to the manufacturer’s instructions. Briefly, EVs were mixed with 1 μM Dil, and the EVs–dye suspension was incubated for 20 min with light avoidance. The excess dye from the labeled EVs was removed by passing through a 100 kDa filter (Microcon YM-100, Millipore), and the EVs pellets were washed three times by resuspending in PBS. The final pellets were resuspended in PBS. Dil-labeled EVs were co-cultured with A549 cells for 6 h, 12 h, 18 h, and 24 h. After treatment, cells were fixed with a 4% paraformaldehyde solution, and nucleus staining was performed using DAPI (Life Technologies, now part of Thermo Fisher Scientific). The uptake was observed by a confocal microscope^24^.

### In vitro cell proliferation assay

The cell proliferation rate was quantified by using MTT (3-(4, 5-dimethyl thiazolyl-2)-2, 5-diphenyltetrazolium bromide) assay. To examine the effect of EVs on A549 cell proliferation, 1 × 10^4^ cells were seeded in a 96-well plate and incubated in exosome-depleted FBS media for 24 h. The purified EVs in different concentrations of 50 μg/ml and 100 μg/ml were directed to cultured cells for 24 h. After the removal of media, 20 μl of MTT reagent (5 mg/ml) was added to each well, and the plate was incubated at 37 °C for 3 h. Formazan crystals were solubilized by adding 100 μl of dimethyl sulfoxide to each well. The optical density at 570 nm was measured using a microplate reader (Epoch-biotek), and the cell proliferation rate was determined. The experiments were performed in triplicates.

### Cell colony-forming assay

A549 cells pre-treated with 50–100 µg/ml EVs for 24 h were collected and seeded into ultralow adhesion 6-well plates (1 × 10^3^ cells/well). The cells were incubated at 37 °C in a humidified atmosphere with 5% CO_2_ in a serum-free medium containing growth factors for 8 days to yield a sufficiently large clone. The medium was changed every 3 days. At the indicated time points, cells were fixed with methanol and stained using crystal violet. Colonies that were visible to the naked eye (consisting of more than 50 cells) were only considered and counted. The results are the mean values of three independent experiments with three replicate plates in each.

### 3-D cell culture assay

A 2 ml agar mixture (DMEM + 0.6% agar) was coated onto a 6-well plate as the bottom layer. A 2 ml agar medium mixture (L-DMEM with 10% FBS + 0.3% agar) containing A549 cells pre-treated with 50-100 µg/ml EVs was added. After the base layer solidified, the top layer was added. The replacement of the medium was carried out at 0.2 ml per well after 1 week of culture. Sphere count was carried out under the microscope after 2 weeks of culture. Each condition has three triplicate wells^25^.

### Scratch assay

A549 cells in the exponential growth phase were grown in 6-well plates until they reached 70–80% confluence. Using a 200 µl plastic pipette tip, the horizontal line across the entire diameter at the bottom of each well was scraped and after that, the scratch was gently washed twice with medium to remove the debris. Cocultured A549 cells in each group were seeded into 6-well plates, respectively, followed by the same treatment as described earlier. The cells migrating into the Scratch were pictured at 0 and 24 h using a microscope (Olympus, Tokyo, Japan). The relative distance between the gaps was captured and measured using an inverted microscope (Olympus)^26^.

### Transwell invasion assay

To assess the ability of EVs to traverse the physical barrier of a transwell membrane, the migration assay was performed on 6-well Transwell® plates (Corning, New York, NY, USA; cat. no. 3422) using inserts with 8-μm pore size membranes. A549 cells were co-cultured with EVs derived from lung cancer bone metastatic patients. A549 cells were diluted 1 × 10^6^ cells/ml and mixed with 50–100 μg/ml EVs in DMEM without FBS. After that cell suspension in each group was added to the upper chamber, and 600 ml DMEM medium containing 20% EVs-free FBS was added to the lower chamber for 24-h incubation in a 37 °C, 5% CO_2_ incubator, followed by 10-min methanol fixation and 10-min 1% crystal violet staining. Five random fields of view were chosen and photographed under an upright microscope^27^.

### Cell cycle analysis

A549 cells were incubated with the 50–100 μg/ml EVs for 24 h. Cells were washed with PBS and harvested by re-suspending in fresh media. Cells were fixed with 70% ethanol to evaluate their effect on the cell cycle using the Muse® Cell Cycle Assay Kit (Part Number: MCH100106) according to the manufacturer's instructions. Results were observed in the percentage of the cells present in the G0-G1 and G2-M phases.

### Real-time PCR (qRT- PCR)

Total RNA was isolated by the TRIzol reagent (Invitrogen, Carlsbad, CA) following the manufacturer’s protocol. A Qiagen cDNA synthesis kit (Cat no: 205411) was used for reverse transcription. Furthermore, cDNA was amplified using Quant studio Real-time PCR System (Applied Biosystems, USA, CA) as per the manufacturer’s protocol (Primer sequences mentioned in Table 2). The relative fold change in expression was calculated using the ΔΔCT method. All experiments were performed in triplicate independently and average CT value was used for further calculations.

### Statistical analysis

Quantitative data are presented as means ± standard error of the mean (SEM). GraphPad Prism software was used for the statistical analyses, version 20.0. The student's t-test and ANOVA were used for data comparison. All experiments were performed in triplicate.

## Supplementary Information

Supplementary Information.

## Data Availability

The datasets generated and/or analyzed during the current study are available in the GEO datasets repository, [GSE54323, GSE32269, GSE175601].
