# Supplementary material for: In vitro model of predicting metastatic ability using tumor derived extracellular vesicles; beyond seed soil hypothesis
Source: Sci Rep. 2022 Nov 24;12:20258. doi: 10.1038/s41598-022-24443-8 (PMC9691738; doi:10.1038/s41598-022-24443-8)

**Supplementary Figure 1**: Internalized health person derived EVs induce malignant transformation of the recipient A549 cells. Fluorescent images of EVs uptake by A549 cells. EVs were stained with DIL dye, and then co-culture with A549 for 24h. Nuclei were stained with DAPI.


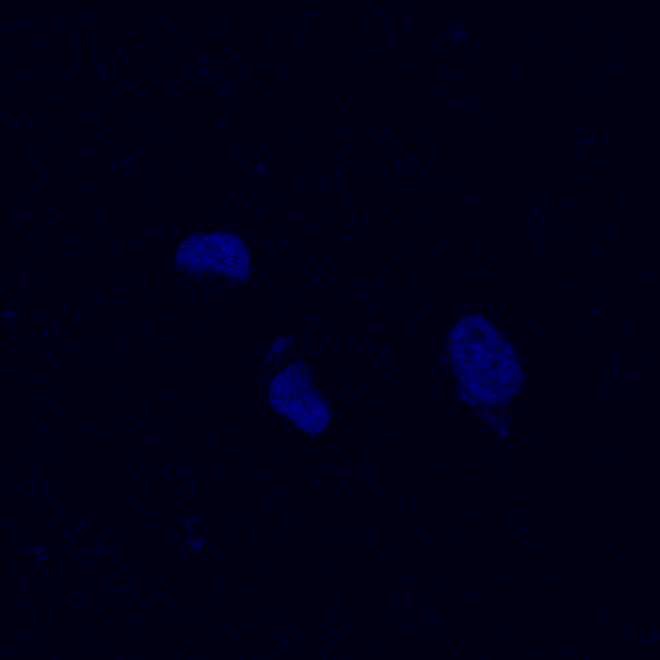

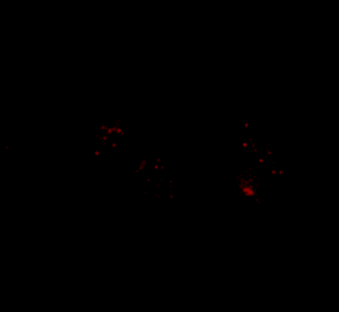

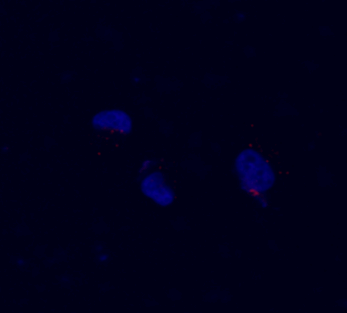


**DAPI**

**Exosomes**

**Merge**

**Supplementary Figure 2**: Full Western Blot Images from Figures. The image was used for Figure 3C.

**CD81**


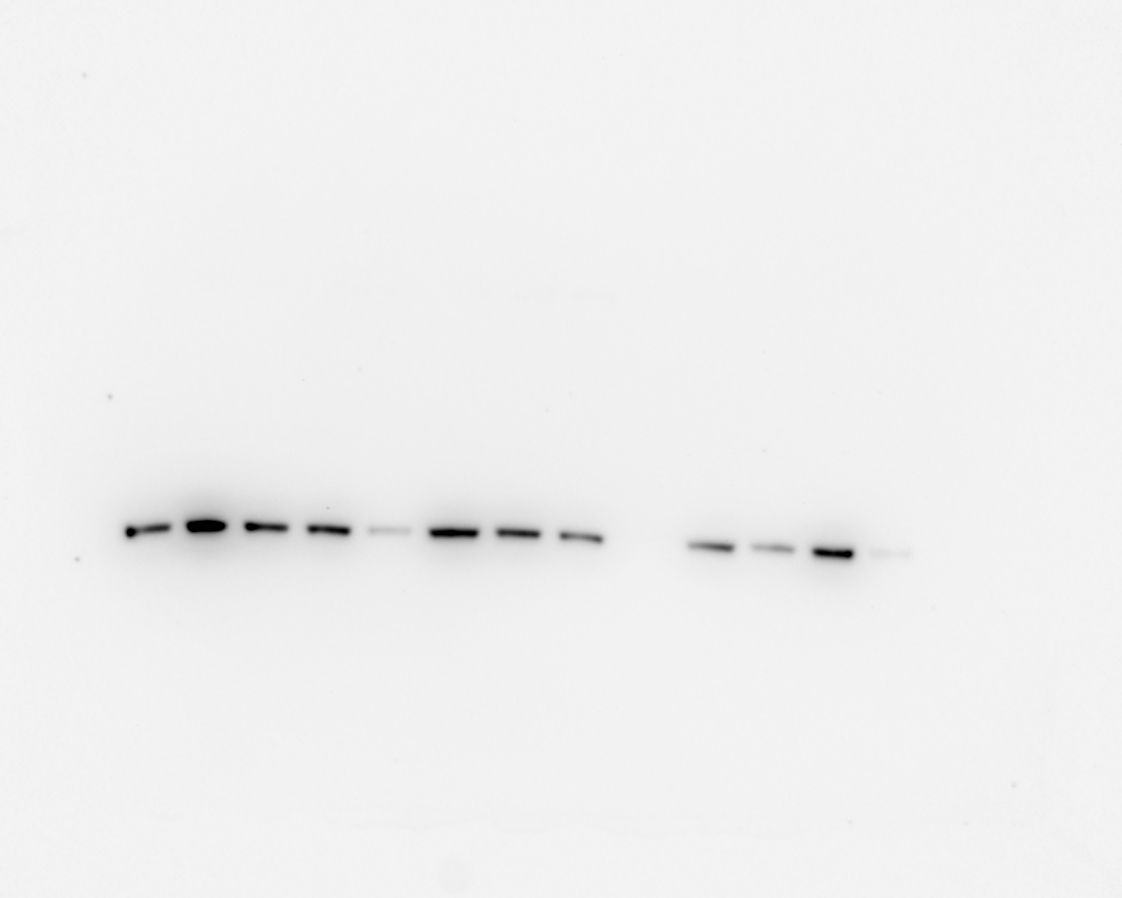


**CD63**


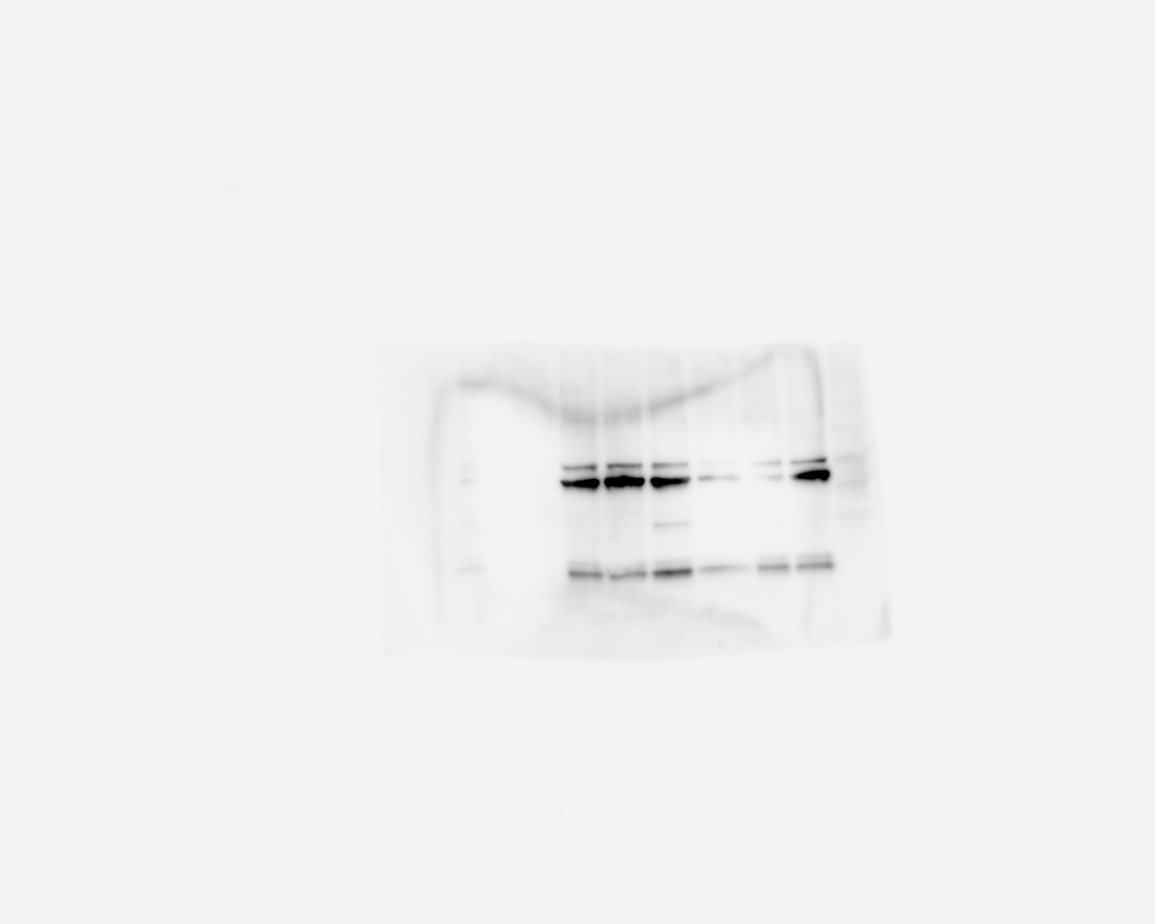

Supplement: Supplementary file 1 — Supplementary Information. [file 41598_2022_24443_MOESM1_ESM.docx]
